# Food Sources Contributing to Intake of Choline and Individual Choline Forms in a Norwegian Cohort of Patients With Stable Angina Pectoris

**DOI:** 10.3389/fnut.2021.676026

**Published:** 2021-05-14

**Authors:** Anthea Van Parys, Therese Karlsson, Kathrine J. Vinknes, Thomas Olsen, Jannike Øyen, Jutta Dierkes, Ottar Nygård, Vegard Lysne

**Affiliations:** ^1^Centre for Nutrition, Department of Clinical Science, University of Bergen, Bergen, Norway; ^2^Department of Internal Medicine and Clinical Nutrition, The Sahlgrenska Academy, University of Gothenburg, Gothenburg, Sweden; ^3^Department of Nutrition, Institute of Basic Medical Sciences, Faculty of Medicine, University of Oslo, Oslo, Norway; ^4^Institute of Marine Research, Bergen, Norway; ^5^Mohn Nutrition Research Laboratory, University of Bergen, Bergen, Norway; ^6^Centre for Nutrition, Department of Clinical Medicine, University of Bergen, Bergen, Norway; ^7^Department of Laboratory Medicine and Pathology, Haukeland University Hospital, Bergen, Norway; ^8^Department of Heart Disease, Haukeland University Hospital, Bergen, Norway

**Keywords:** choline, dietary intake, phosphatidylcholine, FFQ, dietary recommendations

## Abstract

**Background:** Choline is an essential nutrient involved in a wide range of physiological functions. It occurs in water- and lipid-soluble forms in the body and diet. Foods with a known high choline content are eggs, beef, chicken, milk, fish, and selected plant foods. An adequate intake has been set in the US and Europe, however, not yet in the Nordic countries. A higher intake of lipid-soluble choline forms has been associated with increased risk of acute myocardial infarction, highlighting the need for knowledge about food sources of the individual choline forms. In general, little is known about the habitual intake and food sources of choline, and individual choline forms.

**Objective:** Investigate foods contributing to the intake of total choline and individual choline forms.

**Design:** The study population consisted of 1,929 patients with stable angina pectoris from the Western Norway B Vitamin Intervention Trial. Dietary intake data was obtained through a 169-item food frequency questionnaire. Intake of total choline and individual choline forms was quantified using the USDA database, release 2.

**Results:** The geometric mean (95% prediction interval) total choline intake was 287 (182, 437) mg/d. Phosphatidylcholine accounted for 42.5% of total choline intake, followed by free choline (25.8%) and glycerophosphocholine (21.2%). Phosphocholine and sphingomyelin contributed 4.2 and 4.5%, respectively. The main dietary choline sources were eggs, milk, fresh vegetables, lean fish, and bread. In general, animal food sources were the most important contributors to choline intake.

**Conclusion:** This study is, to the best of our knowledge, the first to assess the intake of all choline forms and their dietary sources in a European population. Most choline was consumed in the form of phosphatidylcholine and animal food sources contributed most to choline intake. There is a need for accurate estimates of the dietary intake of this essential nutrient to issue appropriate dietary recommendations.

## Introduction

Choline is an essential nutrient with a variety of biological functions. It is a precursor for the synthesis of phospholipids and the neurotransmitter acetylcholine and a source of methyl groups (1, 2). Choline can be synthesized *de novo* via the hepatic phosphatidylethanolamine N-transferase (PEMT) pathway, however, this route is not sufficient to support biological requirements (3).

As choline has important metabolic functions, both dietary intake and circulating concentrations have been associated with several adverse health effects. Dietary deficiency leads to the development of fatty liver disease, liver and muscle damage, and low choline intake has been associated with cancer, neurodegenerative diseases (1, 4), and low bone mineral density (1, 4, 5). On the other hand, elevated plasma choline levels have been associated with an increased risk of cardiovascular disease (CVD) (4, 6). Contradicting findings have been reported concerning the relationship between choline intake and CVD (7). Recent findings from our group suggest an increased risk of acute myocardial infarction with increased dietary choline intake in patients with suspected CVD (8).

There is some uncertainty regarding the required amount of dietary choline. An adequate intake (AI) value for choline was first set by the US Institute of Medicine (currently known as the National Academies of Medicine, NAM) in 1998 (Table 1) (2). The European Food Safety Authority (EFSA) published the Dietary Reference Values for Choline in 2016. Similar to the NAM, only an AI was set for choline due to a lack of data to determine an estimated average requirement (Table 1) (9). So far, no recommendations have been published for the Nordic countries (10).

**Table 1:** Adequate choline intake (mg/d) in USA and EU^*^.

	**USA**		**EU**
	**Male**	**Female**	
Infant (0–6 months)	125	125	120
Infant (7–12 months)	150	150	160
Children (1–14 years)	200–375	200–375	140–340
Adolescents and adults (≥15 years)	550	400–425	400
Pregnancy		450	480
Lactation		550	520

* *USA recommendations set by the National Academies of Medicine (2). EU recommendations set by the European Food Safety Authority (9)*.

Dietary choline is provided in lipid-soluble forms (phosphatidylcholine or sphingomyelin) or water-soluble forms (free choline, phosphocholine, or glycerophosphocholine). Eggs, beef, chicken, milk and fish, and some plant foods, such as cruciferous vegetables and certain beans, are good sources of choline. Animal products generally contain more choline per weight than plants and contribute most to the intake of the lipid-soluble choline forms (1, 11). Eggs in particular have been shown to make a substantial contribution to total choline intake (12). Further, lecithin (i.e., phosphatidylcholine) is added to many pre-packed foods, which thereby become sources of choline (1).

Choline intake differs between countries as it is dependent on dietary patterns (13) and ethnicity (14). Currently, information on choline intake is mainly available from European and North American countries and has been reviewed by Wiedemann et al. (15) in 2018. Unfortunately, very few studies report on the intake of individual choline forms in addition to total choline intake (15). This could possibly be due to the lack of food composition tables that report on individual choline forms. The USDA database is commonly used for estimation of choline intake (15, 16). Data on dietary choline intake in Norway is scarce (5, 6, 8) and so far, the contribution of different food items to total intake and intake of individual choline forms has not been investigated. Additionally, we were only able to identify one study reporting on contribution of food items to intake of choline forms worldwide (17), emphasizing the knowledge gap regarding this topic.

The aim of this study is to investigate dietary choline intake, including all choline forms, and to map food items contributing to the intake in a Norwegian patient cohort.

## Patients and Methods

### Study Cohort

Between 1999 and 2004, 3,090 adult patients undergoing elective coronary angiography due to suspected coronary artery disease were enrolled in the Western Norway B Vitamin Intervention Trial (WENBIT, NCT00354081) performed at Haukeland University Hospital, Bergen and Stavanger University Hospital, Stavanger in Norway. The WENBIT study was a randomized, double-blind, placebo-controlled prospective secondary prevention study investigating the effect of vitamin B treatment on mortality and cardiovascular outcomes. The study protocol has been described elsewhere (18).

For this study, the source population consisted of the patients from the WENBIT cohort with stable angina pectoris (*n* = 2,573). Exclusion criteria for the current analyses were missing dietary data, including choline intake (*n* = 565), extreme energy intake (i.e., <3,000 kJ or >15,000 kJ for women and <3,300 kJ or >17,500 kJ for men) (*n* = 27) and >10E% from alcohol (*n* = 52), resulting in 1,929 patients eligible for analyses. Key characteristics of the study population are depicted in Table 2.

**Table 2:** Characteristics of the study population.

	**Total population**	**Female**	**Male**
*n* (%)	1,929	390 (20)	1,539 (80)
Age, y	61 (42, 79)	63 (43, 80)	60 (42, 78)
BMI, kg/m^2^	26 (20, 34)	26 (18, 37)	26 (21, 34)
Smokers^a^, *n* (%)	532 (27.6)	100 (25.6)	432 (28.1)
Hypertension, *n* (%)	911 (47.2)	200 (51.3)	711 (46.2)
Diabetes^b^, *n* (%)	592 (30.7)	117 (30.0)	475 (30.9)

*Continuous variables are reported as geometric mean (95% prediction interval), and categorical variables are reported as counts (%)*.

a *Defined according to self-reporting smoking habits and serum cotinine levels >85 nmol/L at baseline*.

b *Defined according to pre-existing diagnosis, HbA1c >6.5%, fasting blood glucose ≥7 mmol/L or non-fasting blood glucose ≥11.1 mmol/L*.

The study was carried out according to the Declaration of Helsinki and approved by the Regional Committee for Medical Health Research Ethics and the Norwegian Data Inspectorate. All participants provided written informed consent.

### Dietary Assessment

A 169-item food frequency questionnaire (FFQ) was given to the patients at the first study visit, filled out by the patients, and returned at the 1-month follow-up visit or returned by mail to the study center. The administered FFQ was an adaptation of an FFQ developed at the Department of Nutrition, University of Oslo designed to obtain information on habitual food intake of the Norwegian population over the past year. Portion sizes were given as units (e.g., slices, pieces, etc.) or household measures. Depending on the food item, the frequency of consumption was given per day, week, month, or never consumed. Questions on vitamin and supplement use were included, however, there were no specific questions regarding choline supplementation. A software system developed at the Department of Nutrition, University of Oslo (Kostberegningssystem, version 3.2, University of Oslo, Norway) was used to calculate energy and nutrient intakes.

### Choline Composition Data

Choline composition data are currently not available within the Norwegian food composition database (19). Choline content of food items was therefore quantified using the U.S. Department of Agriculture (USDA) Database for Choline Content of Common Foods, release 2 (11). This database contains the choline content of over 630 food items, analyzed using liquid chromatography-electrospray ionization-isotope dilution mass spectrometry (LC-ESI-MS) (11). Information on total choline content is provided both in the database and in this study as the sum of the five choline forms - free choline, glycerophosphocholine, phosphocholine, phosphatidylcholine, and sphingomyelin. The choline content of food items included in the FFQ but missing in the USDA database was estimated using nutritionally equivalent foods. For multi-component foods (e.g., dishes, fast foods), choline content was calculated for each ingredient in the FFQ recipe.

Food entries were sorted into 41 subcategories based on nutrient similarities. These categories were gathered into 28 main categories. Finally, the main categories were gathered into 10 food groups. A detailed overview is shown in Supplementary Table 1.

### Statistical Analyses

Continuous variables are reported as geometric means (95% prediction interval [PI]). The 95% PI renders the limits of the interval as defined by [(geometric mean)/(geometric standard deviation)^2^, (geometric mean) × (geometric standard deviation)^2^]. The residual method was used to adjust choline intake for reported energy intake. Other dietary variables were energy-adjusted using the density method and are reported as energy % (E%) or g/1,000 kcal.

The percent contribution of each (sub) category to total choline intake and intake of individual choline forms was calculated using the following formula: [(choline provided by the food (sub) category/Total choline from all food (sub) categories)]^*^100.

In accordance with the Strengthening the Reporting of Observational studies in Epidemiology (STROBE) checklist and statement (20), we chose not to report *p*-values.

All statistical analyses were performed using R version 3.6.1 [The R Foundation for Statistical Computing, Vienna, Austria) and the packages within the *Tidyverse (version 1.3.0)* (21) (*broom (version 0.5.6), dplyr (version 0.8.5), forcats (version 0.5.0), ggplot2 (version 3.3.0), magrittr (version 1.5), purr (version 0.3.4), rlang (version 0.4.5), stringr (version 1.4.0), tidyr (version 1.0.2)*].

## Results

The mean (95% PI) age of the participants was 61 (42, 79) years and 80% were men. The participants consumed on average 1996 (982, 3,512) kcal per day of which 49.5 E% came from carbohydrates, 16.8 E% from protein, and 30.9 E% from fat. An overview of the dietary intake in the study population is provided in Table 3.

**Table 3:** Dietary intake in the total study population and across genders.

	**Total population**	**Female**	**Male**
*n* (%)	1,929	390 (20)	1,539 (80)
Energy intake (kcal)	1,996 (983, 3,512)	1,548 (834, 2,861)	2,128 (1,196, 3,594)
Carbohydrates (E%)	48.7 (36.7, 60.8)	49.7 (37.5, 61.3)	48.5 (36.4, 60.4)
Protein (E%)	16.5 (12.1, 22.3)	17.1 (12.8, 23.1)	16.4 (12.0, 22.0)
Fat (E%)	31.5 (21.6, 43.1)	30.7 (21.0, 42.5)	31.7 (21.7, 43.2)
MUFA (E%)	10.1 (6.6, 14.3)	9.8 (6.6, 13.7)	10.2 (6.6, 14.4)
PUFA (E%)	7.0 (4.2, 11.5)	6.5 (4.1, 10.6)	7.1 (4.3, 11.8)
SFA (E%)	11.5 (7.1, 17.7)	11.5 (7.1, 18.5)	11.5 (7.0, 17.4)
Alcohol (E%)	0.2 (0.0, 7.5)	0.0 (0.0, 5.6)	0.3 (0.0, 7.7)
Dairy	126 (17, 413)	135 (26, 445)	124 (16, 403)
Drinks	552 (202, 1,359)	658 (232, 1,562)	529 (197, 1,227)
Eggs	5 (0, 24)	6 (0, 26)	5 (0, 24)
Fats	13 (2, 32)	11 (2, 30)	13 (2, 32)
Fish	45 (10, 119)	46 (10, 124)	45 (11, 119)
Fruit	98 (16, 339)	122 (17, 376)	93 (15, 330)
Grain products	118 (62, 187)	115 (58, 185)	118 (63, 188)
Meat	49 (16, 105)	48 (16, 100)	50 (16, 106)
Other	40 (9, 118)	38 (9, 108)	40 (10, 118)
Vegetables	153 (58, 361)	178 (58, 412)	147 (58, 332)

*All dietary intakes are presented as geometric mean (95% prediction interval) and as g/1,000 kcal unless specified otherwise. E% indicates energy percent; MUFA, monounsaturated fatty acids; PUFA, polyunsaturated fatty acids; SFA, saturated fatty acids*.

Table 4 shows the energy-adjusted self-reported daily choline intake among the study participants. The geometric mean energy-adjusted total choline intake in the population was 287 (182, 437) mg/d. Women seemed to have a slightly higher choline intake compared to men. Phosphatidylcholine was the major contributor (42.5%) followed by free choline and glycerophosphocholine (respectively 25.8 and 21.2% of total choline intake). Finally, sphingomyelin and phosphocholine contributed to the total intake with 4.5 and 4.2% respectively. Reported energy-adjusted intakes at, or above, the AI of 400 mg/d as defined by EFSA were achieved in only 5.5% of the study population.

**Table 4:** Mean energy-adjusted daily reported choline intake in the study population and across sex.

	**Total population**	**% of total choline**	**Female**	**Male**
*n* (%)	1,929		390 (20)	1,539 (80)
Total choline, mg/d	287 (182, 436)	-	294 (216, 435)	285 (178, 439)
Free choline, mg/d	74 (49, 114)	25.8	76 (53, 110)	74 (48, 116)
Glycerophosphocholine, mg/d	61 (24, 128)	21.2	62 (31, 109)	61 (23, 132)
Phosphatidylcholine, mg/d	122 (67, 209)	42.5	127 (78, 216)	121 (66, 206)
Phosphocholine, mg/d	13 (5, 26)	4.2	14 (7, 32)	12 (5, 25)
Sphingomyelin, mg/d	13 (7, 22)	4.5	13 (8, 21)	13 (7, 22)

*Intakes are reported as geometric means (95% prediction interval)*.

**Figure 1:**
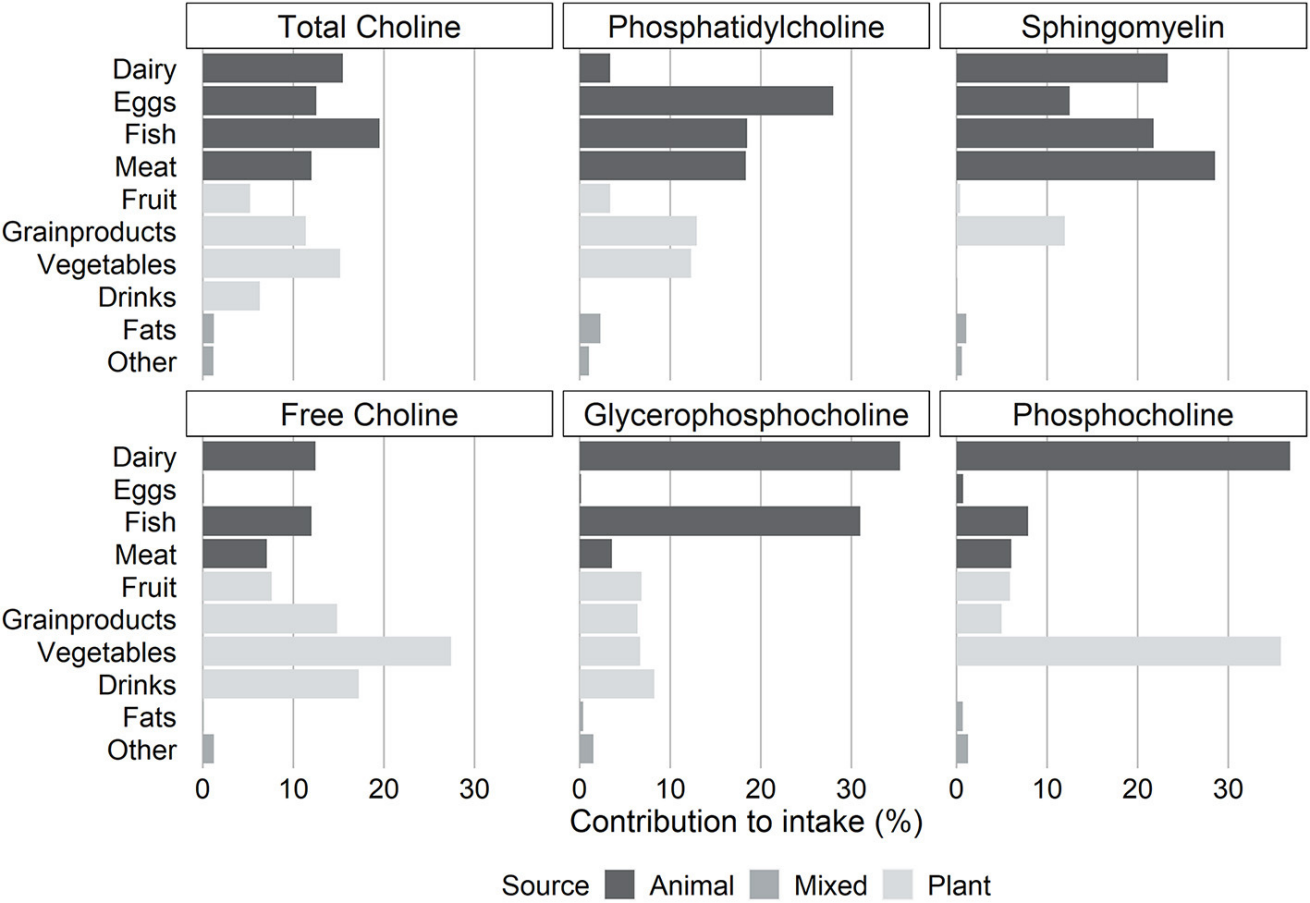
Contribution of food groups to the intake of total choline and individual choline forms.

In this population, the main choline source was fish, followed by dairy, vegetables, eggs, and meat which accounted in total for about 75% of the total choline intake (Figure 1). Phosphatidylcholine was mainly obtained from eggs (28.0%), fish (18.5%), and meat (18.3%). The contribution of these foods combined provided 65% of the total phosphatidylcholine intake. The food category contributing the most to sphingomyelin in the diet was meat (28.5%), followed by dairy (23.3%) and fish (21.7%). Free choline was mainly obtained from vegetables, drinks, grain products, and dairy. Dairy was the main source for both glycerophosphocholine and phosphocholine in our study cohort and accounted for respectively 35.4 and 36.8% of their intake.

Table 5 depicts a more detailed picture of dietary choline sources showing food categories, instead of the larger food groups, contributing to dietary intake of total choline. Eggs contributed most to total choline intake in this population. Additionally, animal-based foods made up seven out of 10 food categories providing most total choline.

**Table 5:** Primary food categories contributing to total choline intake in the study population.

**Rank**	**Food category**	**Contribution, %**	**Cumulative contribution**
1	Eggs	12.6	12.6
2	Milk	12.1	24.7
3	Fresh vegetables	9.2	33.9
4	Lean fish	8.3	42.2
5	Bread	7.3	49.5
6	Fish products	6.3	55.8
7	Potatoes	5.5	61.3
8	Meat products	5.4	66.7
9	Fresh meat	5.1	71.8
10	Coffee	3.9	75.7

The main food categories contributing to intake of the individual choline forms are shown in Supplementary Tables 2a–e. Fresh vegetables, bread, coffee, and potatoes supplied half of the dietary free choline intake in our study population. Glycerophosphocholine was primarily obtained from milk and different fish sources, while phosphocholine was mainly acquired through intake of milk, fresh vegetables, and potatoes. The main source of the lipid-soluble phosphatidylcholine was eggs, contributing with 28%. Fresh meat, eggs, milk, and fish products provided half of the ingested sphingomyelin. A full overview of all food groups, categories, and subcategories contributing to choline intake is provided in Supplementary Table 3.

**Figure 2:**
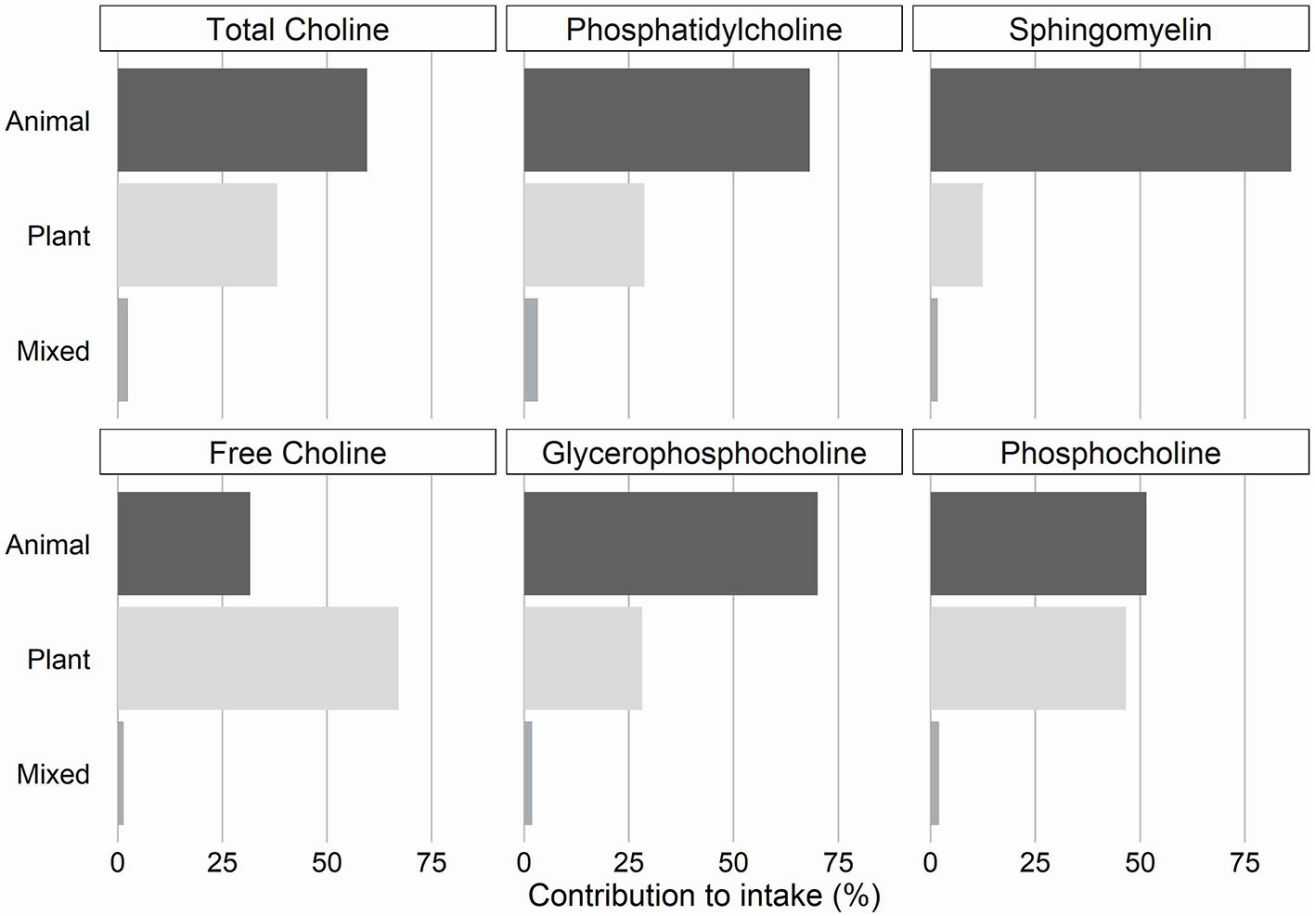
Contribution of animal, plant or mixed food sources to intake of total choline and individual choline forms.

Total choline and all individual choline forms, except for free choline, were mainly obtained from animal-based food sources in this study population (Figure 2).

## Discussion

This study aimed to investigate food items contributing to the intake of total choline and individual choline forms. Eggs, milk, fresh vegetables, lean fish, and bread were the main contributors to total choline intake. Choline was mainly consumed in the form of phosphatidylcholine. In general, animal food sources were the most important contributors to choline intake. To our knowledge, this is the first study to assess dietary sources of choline and intake of all choline forms in a European population.

### Dietary Sources of All Choline Forms

Eggs contain the highest amount of choline per weight (11) and contribute most to total choline intake in this study. Other good choline sources such as meat, fish, and milk also ranked highest among choline contributors. Similar findings have been reported in other Western cohorts (14, 17, 22–26). Differences in the contribution of food groups between cohorts might be due to differences in dietary patterns or a different definition of the food groups. However, there was a consensus between the reported studies on the major food groups contributing being eggs, milk, meat, and fish.

A limited amount of studies reports on the intake of individual choline forms. The distribution of the intakes of the individual choline forms observed in this cohort accords with the distribution reported in other Western cohorts (15, 24, 25, 27). The lipid-soluble choline form phosphatidylcholine accounted for around half of the total ingested choline in this cohort. This is not surprising since 60% of total choline was obtained from animal products, which in general contain more choline per unit weight than plants and contain mainly lipid-soluble choline forms (1, 11). We could only identify one study describing the contribution of food items to every individual choline form (17). Similar to our findings, lipid-soluble choline forms were mainly obtained from animal-derived food items, while the contribution of plant foods was larger for the water-soluble forms. Top-ranked contributing food items were also similar for all individual choline forms.

### Total Choline Intake

Dietary choline intake has mainly been studied in European and North-American cohorts (15). Based on food consumption data from the EFSA European Comprehensive Food Consumption Database, Vennemann et al. reported a self-reported choline intake ranging from 357 to 468 mg/d for adult men and from 293 to 374 mg/d for adult women in Europe (16). This data was obtained from 10 nationwide surveys in eight different European countries. These values are similar to the dietary choline intake reported in several studies conducted in the USA (14, 17, 22, 23, 26, 28, 29), Canada (25, 30) and New-Zealand (24) where reported intake ranged from 312 to 421 mg/d in adult men and from 258 to 314 mg/d in adult women. The self-reported dietary choline intake in our population was low compared to these values. It is possible that choline intake is lower in Norway compared to other Western countries since choline consumption is dependent on individual dietary patterns. Our population was generally older and our data was collected at an earlier time point compared to the mentioned studies, which might explain this discrepancy. Moreover, it has been shown that race and ethnicity influence choline intake (14, 23), which might explain the discrepancies further. However, the Norwegian dietary habits are quite similar to those of other Western countries and it would, therefore, be unlikely that this causes the lower intake. Notably, we used an FFQ that was not validated for choline intake. Therefore, we were unable to evaluate how well it assesses the actual choline intake. Further, studies have used different versions of the USDA database to assess to choline content of foods, which leads to variation in estimates of choline intake. Also, some studies adjusted for energy intake, while others did not. Finally, it has to be taken into account that comparing dietary choline intake between studies must be done with caution due to different methods used to assess dietary habits.

Notably, the total dietary choline intake in our cohort, as in many other studies (15), was below the recommended European and American AI. The self-reported nature of the dietary choline data may have caused an underestimation of actual choline intake due to underreporting. Fischer et al. (28) found that self-reported 3-day weighed food records significantly underestimated daily choline intake compared to the measured choline content in the diet. Additionally, FFQs are subject to social desirability bias meaning that participants tend to overreport food items that are considered “healthy.” This could have led to underreporting of “less healthy” food items in this population such as eggs and red meat, which are rich in choline. Egg consumption was discouraged in Norway at the time of data collection which may have lead to underreporting or a true lower egg intake. It also has to be taken into account that the recommended AI of both NAM and EFSA is based on few data. The values set by the NAM for adults are based on a single study performed in males, whereas the values for children were mathematically extrapolated from these adult values (2). EFSA based its estimates on 12 national surveys undertaken in nine European countries (31). Both institutions agree that there is insufficient data to establish average requirements and population reference intakes and therefore only report an AI (2, 31). The lack of data could be attributed to the lack of food composition databases to estimate dietary choline intake. Additionally, folic acid fortification in grains in the US improved folate status in this population. This may reduce choline requirements since folate can be used for remethylation of homocysteine, thereby sparing choline (32) and thus influence dietary requirements set by NAM. Finally, given the definition of an AI, it is not possible to draw any conclusion about the adequacy of choline intake in this study population.

### Strengths and Limitations

Several limitations of our study should be acknowledged. First, the Norwegian food composition table does not include values for choline (19). Since choline composition data of European foods is also non-existent, we based our calculation of the choline content of food items on the USDA database (11). The choline content of food items in this database might not always reflect the true choline content of consumed food items in this study population. Especially local foods, which may not be typically consumed in a North American diet needed to be substituted with similar foods with a known choline content. Choline content can also differ due to variations in recipes used by the manufactures or due to differences in choline content of the individual ingredients (16). Additionally, it is not unlikely that the nutritional content of animal food items is influenced by factors such as choline consumption of the animal, season and geographical location, and variation between and within animals, all of which can contribute to discrepancies between estimated and actual dietary choline intake.

Secondly, the administered FFQ was not validated for choline intake, meaning that we were not able to evaluate its ability to capture actual choline intake. The reported choline intakes should therefore be interpreted with caution. It also did not include information on the food preparation method, which influences the choline content (11, 13). General disadvantages of an FFQ also apply here and include failure to report intake of non-included items due to the fixed-food list and recall bias (33).

Finally, this population of, mainly older, male, cardiovascular patients is not representative of the general population. Additionally, a high percentage of the study population suffered from chronic conditions such as hypertension (almost 50%) and diabetes (30%). Medication use (e.g., statins, aspirin, β-blockers) was also very common in this study cohort (data not shown). The population has been shown to be representative of a general CVD disease population (18), and the results may therefore lack external validity outside such populations. Moreover, their diagnosis might have influenced their true and reported usual dietary intake as these patients may have received dietary advice. Especially consumption of animal food items, which is discouraged in these patients, could have been affected, leading to both reduced intake and underreporting of total choline intake (34).

The strength of this study is that intake of all choline forms, which is heavily understudied, was estimated in a large cohort. Moreover, using an FFQ for dietary assessments avoids day-to-day variations and represents usual long-term intake. This allowed us to collect data on food items that are less frequently consumed. Finally, an FFQ captures usual long-term dietary intake, allowing us to evaluate the main food items contributing to choline intake (33).

## Conclusion

In conclusion, this study is the first to assess the intake of all choline forms and their dietary contributors in a European population. We found that the main contributors to total choline intake were eggs, milk, fresh vegetables, lean fish, and bread. Most choline was consumed in the form of phosphatidylcholine and animal food sources were the most important contributors to intake off all choline forms except free choline. More research is needed to better understand dietary choline requirements. There is an urgent need for a Norwegian database to more accurately estimate the dietary intake of this essential nutrient. Better understanding of dietary choline intake is essential to improve insight in its association with health outcomes.

## Data Availability Statement

The data analyzed in this study is subject to the following licenses/restrictions: The WENBIT dataset is not publicly available. Requests to access these datasets should be directed to ottar.kjell.nygard@helse-bergen.no.

## Ethics Statement

The studies involving human participants were reviewed and approved by Regional Committee for Medical Health Research Ethics. The patients/participants provided their written informed consent to participate in this study.

## Author Contributions

KV and TK calculated the dietary choline intake. AVP analyzed the data and wrote the paper. All authors read and approved the final manuscript.

## Conflict of Interest

The authors declare that the research was conducted in the absence of any commercial or financial relationships that could be construed as a potential conflict of interest.
