# Supplementary material for: Food Sources Contributing to Intake of Choline and Individual Choline Forms in a Norwegian Cohort of Patients With Stable Angina Pectoris
Source: Front Nutr. 2021 May 14;8:676026. doi: 10.3389/fnut.2021.676026 (PMC8160433; doi:10.3389/fnut.2021.676026)
Supplement: Supplementary file 1 [file Data_Sheet_1.docx]

Supplementary Material

| **Supplementary Table 1:** Grouping of food items into subcategories, categories and food groups | | | |
| --- | --- | --- | --- |
| **Food group** | **Category** | **Subcategory** | **Food items** |
| Dairy | Milk | Whole milk | Whole milk |
|  |  | Low-fat milk | Low-fat milk  Skimmed milk  Unspecified milk |
|  | Cheese | White cheese | White cheese >27% fat  White cheese 27% fat  White cheese 16% fat |
|  |  | Brown cheese | Brown cheese |
|  | Other dairy | Yogurt | Plain yogurt  Fruit yogurt low fat  God morgen forest fruits |
|  |  | Cream | Cream 10% fat  Sour cream |
|  |  | Ice cream | Ice cream |
|  |  | Other dairy products | Pudding |
| Drinks | Coffee |  | Boiled coffee  Instant coffee  Filter coffee  Decaf |
|  | Tea |  | Black tea  Herbal tea |
|  | Soda | Soda sugar | Soda with sugar  Juice with sugar |
|  |  | Soda light | Soda light  Soda with sweetener |
|  | Alcohol | Beer | Alcohol-free beer  Pilsner beer |
|  |  | Wine | Wine |
|  |  | Liquor | Liquor |
| Eggs |  |  |  |
| Fats | Margarine |  | Soya margarine  Margarine mixture  Other margarine  Light margarine |
|  | Butter |  | Butter  Butter-margarine mixture  Unspecified butter |
|  | Other fats |  | Mayonnaise  Remoulade  Dressing  Mayonnaise in salads |
| Fish | Lean fish |  | Cod  Pollock  Lean and half-fatty fish |
|  | Fatty fish |  | Salmon  Trout  Herring  Mackerel |
|  | Fish products | Minced fish | Mined fish products |
|  |  | Breaded fish | Breaded fish  Deep-fried fish |
|  |  | Fish spread | Mackerel  Sardines  Herring  Caviar |
|  | Shellfish |  | Shrimp  Crab |
| Fruit | Fresh fruit |  | Citrus fruits  Apples  Pears  Bananas  Grapes  Exotic fruits  Blueberries  Strawberries  Cloudberries  Unspecified fruit |
|  | Canned fruit |  | Canned apricots |
|  | Other fruit | Juice | Orange juice  Other juice  Unfiltered juice  Nectar |
|  |  | Jam | Jam |
| Grain products | Bread | White bread | White bread  Bread <50% whole wheat White rolls  Rolls <50% whole wheat |
|  |  | Wholegrain bread | Bread >50% whole wheat  Rolls >50% whole wheat  “Birkebeiner” Bread |
|  |  | Other bread | “Lefse”  Chapatti  Taco  Flatbread  Crispbread |
|  | Pastries | Buns | Waffles  “School bun”  Danish pastry |
|  |  | Cookies | Cookies |
|  |  | Other pastries | Cakes with filling  Cakes made with lard |
|  | Other grains | Rice | Rice |
|  |  | Pasta | Pasta |
|  |  | Pizza | Pizza |
|  |  | Cereal | Cereal  Oatmeal |
| Meat | Meat products | Sausages | Wiener sausage  Cooked sausage |
|  |  | Liver spread | Liver spread  Liver spread 13% fat |
|  |  | Meat spread | Light meat spread  Salami  Saveloy |
|  | Fresh meat | Poultry | Poultry |
|  |  | Venison | Venison |
|  |  | Other fresh meat | Minced meat dish  Minced meat products |
|  | Other meat |  | Unspecified meat |
| Other | Sugar and sweets | Sugar | Sugar  Other sweeteners Energy-free sweeteners |
|  |  | Sweet spread | Honey Sweet spread |
|  |  | Chocolate | Chocolate |
|  |  | Candy | Candy |
|  | Snacks | Chips | Potato chips |
|  |  | Nuts and seeds | Nuts and seeds |
|  |  | Other snacks | Other snacks |
| Vegetables | Potatoes |  | Fresh potatoes  French fries |
|  | Fresh vegetables |  | Carrots  Kohlrabi  Cabbage  Cauliflower  Broccoli  Onion  Leek  Tomato  Bell pepper  Kale  Spinach  Mushrooms  Avocado  Corn Vegetable mix Vegetables in stews Vegetable spread |
|  | Canned vegetables |  | Canned beans  Pickled vegetables  Tomato ketchup  Other canned vegetables |

| **Supplementary Table 2a:** Primary food categories contributing to the intake of free choline | | | |
| --- | --- | --- | --- |
| **Rank** | **Food category** | **Contribution, %** | **Cumulative contribution** |
| 1 | Fresh vegetables | 16.0 | 16.0 |
| 2 | Bread | 11.6 | 27.6 |
| 3 | Coffee | 11.5 | 39.1 |
| 4 | Potatoes | 10.7 | 49.8 |
| 5 | Milk | 9.5 | 59.3 |
| 6 | Fresh fruit | 5.6 | 64.9 |
| 7 | Lean fish | 5.5 | 70.4 |
| 8 | Alcohol | 5.0 | 75.4 |
| 9 | Meat products | 4.8 | 80.2 |
| 10 | Fish products | 3.8 | 84.0 |

| **Supplementary Table 2b:** Primary food categories contributing to the intake of glycerophosphocholine | | | |
| --- | --- | --- | --- |
| **Rank** | **Food category** | **Contribution, %** | **Cumulative contribution** |
| 1 | Milk | 30.1 | 30.1 |
| 2 | Lean fish | 13.8 | 43.9 |
| 3 | Fish products | 8.9 | 52.8 |
| 4 | Fatty fish | 7.9 | 60.7 |
| 5 | Bread | 4.8 | 65.5 |
| 6 | Potatoes | 4.4 | 69.9 |
| 7 | Coffee | 4.4 | 74.3 |
| 8 | Other dairy | 4.3 | 78.6 |
| 9 | Alcohol | 3.5 | 82.1 |
| 10 | Fresh fruit | 3.5 | 85.6 |

| **Supplementary Table 2c:** Primary food categories contributing to the intake of phosphocholine | | | |
| --- | --- | --- | --- |
| **Rank** | **Food category** | **Contribution, %** | **Cumulative contribution** |
| 1 | Milk | 31.1 | 31.1 |
| 2 | Fresh vegetables | 27.1 | 58.2 |
| 3 | Potatoes | 8.3 | 66.5 |
| 4 | Other dairy | 4.4 | 70.9 |
| 5 | Fresh meat | 4.0 | 74.9 |
| 6 | Fresh fruit | 3.6 | 78.5 |
| 7 | Fish products | 3.5 | 82.0 |
| 8 | Lean fish | 3.4 | 85.4 |
| 9 | Bread | 3.1 | 88.5 |
| 10 | Other fruit | 2.0 | 90.5 |

| **Supplementary Table 2d:** Primary food categories contributing to the intake of phosphatidylcholine | | | |
| --- | --- | --- | --- |
| **Rank** | **Food category** | **Contribution, %** | **Cumulative contribution** |
| 1 | Eggs | 28.0 | 28.0 |
| 2 | Fresh meat | 8.2 | 36.2 |
| 3 | Fresh vegetables | 8.1 | 44.3 |
| 4 | Lean fish | 7.7 | 52 |
| 5 | Meat products | 7.6 | 59.6 |
| 6 | Bread | 6.7 | 66.3 |
| 7 | Fish products | 6.1 | 72.4 |
| 8 | Pastries | 4.8 | 77.2 |
| 9 | Potatoes | 3.8 | 81 |
| 10 | Shellfish | 3.6 | 84.6 |

| **Supplementary Table 2e:** Primary food categories contributing to the intake of sphingomyelin | | | |
| --- | --- | --- | --- |
| **Rank** | **Food category** | **Contribution, %** | **Cumulative contribution** |
| 1 | Fresh meat | 15.7 | 15.7 |
| 2 | Eggs | 12.5 | 28.2 |
| 3 | Milk | 12.3 | 40.5 |
| 4 | Fish products | 10.4 | 50.9 |
| 5 | Meat products | 9.9 | 60.8 |
| 6 | Bread | 8.4 | 69.2 |
| 7 | Cheese | 7.1 | 76.3 |
| 8 | Lean fish | 4.4 | 80.7 |
| 9 | Other dairy | 3.9 | 84.6 |
| 10 | Fatty fish | 3.5 | 88.1 |

| **Supplementary Table 3: Overview of the contribution of various food items to dietary intake of total choline and individual choline forms** | | | | | | | | | | | | |
| --- | --- | --- | --- | --- | --- | --- | --- | --- | --- | --- | --- | --- |
|  | | | |  |  | **Water-soluble forms** | | |  | **Lipid-soluble forms** | | |
| Contributing food items (%) | | | | **Total choline** |  | **Free choline** | **Glycerophospho-choline** | **Phosphocholine** |  | **Phosphatidylcholine** | **Sphingomyelin** |  |
| Dairy | | | | 15.5 |  | 12.4 | 35.4 | 36.8 |  | 3.4 | 23.3 |  |
|  | Milk | | | 12.1 |  | 9.4 | 30.0 | 31.0 |  | 1.7 | 12.3 |  |
|  |  | | Whole milk | 1.0 |  | 1.0 | 2.2 | 3.1 |  | 0.1 | 1.1 |  |
|  |  | | Low-fat milk | 11.1 |  | 8.4 | 27.8 | 27.9 |  | 1.6 | 11.2 |  |
|  | Cheese | | | 1.4 |  | 1.4 | 1.0 | 1.4 |  | 1.0 | 7.1 |  |
|  |  | | White cheese | 1.0 |  | 0.9 | 0.9 | 1.4 |  | 0.7 | 4.8 |  |
|  |  | | Brown cheese | 0.4 |  | 0.4 | 0.1 | 0.0 |  | 0.4 | 2.3 |  |
|  | Other dairy | | | 2.1 |  | 1.6 | 4.3 | 4.4 |  | 0.6 | 3.9 |  |
|  |  | | Yogurt | 1.1 |  | 0.7 | 2.5 | 2.9 |  | 0.3 | 1.9 |  |
|  |  | | Cream | 0.4 |  | 0.3 | 0.7 | 0.5 |  | 0.1 | 1.0 |  |
|  |  | | Ice cream | 0.5 |  | 0.3 | 1.0 | 0.7 |  | 0.2 | 1.0 |  |
|  |  | | Other products | 0.2 |  | 0.3 | 0.3 | 0.3 |  | 0.0 | 0.0 |  |
| Drinks | | | | 6.3 |  | 17.2 | 8.2 | 0.0 |  | 0.0 | 0.0 |  |
|  | Coffee | | | 3.9 |  | 11.5 | 4.4 | 0.0 |  | 0.0 | 0.0 |  |
|  | Tea | | | 0.2 |  | 0.7 | 0.0 | 0.0 |  | 0.0 | 0.0 |  |
|  | Soda | | | 0.1 |  | 0.0 | 0.4 | 0.0 |  | 0.0 | 0.0 |  |
|  | Alcohol | | | 2.1 |  | 5.0 | 3.5 | 0.0 |  | 0.0 | 0.0 |  |
| Eggs | | | | 12.6 |  | 0.1 | 0.1 | 0.7 |  | 28.0 | 12.5 |  |
| Fats | | | | 1.2 |  | 0.1 | 0.4 | 0.7 |  | 2.3 | 1.1 |  |
|  | Margarine | | | 0.5 |  | 0.0 | 0.2 | 0.3 |  | 1.1 | 0.0 |  |
|  | Butter | | | 0.2 |  | 0.0 | 0.1 | 0.4 |  | 0.2 | 0.5 |  |
|  | Other fats | | | 0.5 |  | 0.0 | 0.2 | 0.0 |  | 1.0 | 0.6 |  |
| Fish | | | | 19.5 |  | 12.0 | 31.0 | 7.9 |  | 18.5 | 21.7 |  |
|  | Lean fish | | | 8.3 |  | 5.5 | 13.8 | 3.4 |  | 7.7 | 4.4 |  |
|  | Fatty fish | | | 3.1 |  | 2.5 | 7.9 | 0.6 |  | 1.2 | 3.5 |  |
|  | Fish products | | | 6.3 |  | 3.8 | 8.9 | 3.5 |  | 6.1 | 10.4 |  |
|  |  | | Minced fish | 1.1 |  | 1.0 | 1.8 | 0.9 |  | 0.8 | 1.1 |  |
|  |  | | Breaded fish | 0.4 |  | 0.3 | 0.6 | 0.2 |  | 0.4 | 0.1 |  |
|  |  | | Fish spread | 4.8 |  | 2.5 | 6.5 | 2.5 |  | 5.0 | 9.1 |  |
|  | Shellfish | | | 1.9 |  | 0.1 | 0.5 | 0.4 |  | 3.6 | 3.5 |  |
| Fruit | | | | 5.2 |  | 7.6 | 6.8 | 5.9 |  | 3.4 | 0.4 |  |
|  | Fresh fruit | | | 3.4 |  | 5.6 | 3.5 | 3.6 |  | 2.4 | 0.2 |  |
|  | Canned fruit | | | 0.2 |  | 0.0 | 0.2 | 0.3 |  | 0.2 | 0.2 |  |
|  | Other fruit | | | 1.7 |  | 2.0 | 3.1 | 2.0 |  | 0.9 | 0.0 |  |
| Grain products | | | | 11.4 |  | 14.9 | 6.4 | 5.0 |  | 13.0 | 12.0 |  |
|  | | Bread | | 7.3 |  | 11.6 | 4.8 | 3.1 |  | 6.7 | 8.4 |  |
|  | |  | White bread | 2.5 |  | 4.0 | 1.6 | 1.3 |  | 2.2 | 2.5 |  |
|  | |  | Wholegrain bread | 4.4 |  | 7.1 | 2.7 | 1.6 |  | 4.0 | 5.4 |  |
|  | |  | Other bread | 0.5 |  | 0.6 | 0.6 | 0.2 |  | 0.4 | 0.4 |  |
|  | | Pastries | | 2.6 |  | 1.0 | 0.7 | 0.8 |  | 4.8 | 2.2 |  |
|  | |  | Buns | 2.0 |  | 0.7 | 0.5 | 0.6 |  | 3.7 | 1.7 |  |
|  | |  | Cookies | 0.1 |  | 0.2 | 0.0 | 0.0 |  | 0.1 | 0.0 |  |
|  | |  | Other pastries | 0.5 |  | 0.2 | 0.1 | 0.1 |  | 1.0 | 0.5 |  |
|  | | Other grains | | 1.5 |  | 2.2 | 0.9 | 1.1 |  | 1.6 | 1.4 |  |
|  | |  | Rice | 0.1 |  | 0.2 | 0.3 | 0.0 |  | 0.1 | 0.0 |  |
|  | |  | Pasta | 0.2 |  | 0.5 | 0.1 | 0.0 |  | 0.2 | 0.0 |  |
|  | |  | Pizza | 0.6 |  | 0.9 | 0.2 | 0.8 |  | 0.5 | 0.9 |  |
|  | |  | Cereal | 0.6 |  | 0.6 | 0.3 | 0.3 |  | 0.8 | 0.5 |  |
| Meat | | | | 12.0 |  | 7.1 | 3.6 | 6.1 |  | 18.3 | 28.5 |  |
|  | | Meat products | | 5.4 |  | 4.8 | 1.5 | 1.8 |  | 7.6 | 9.9 |  |
|  | |  | Sausages | 2.2 |  | 3.9 | 0.2 | 0.9 |  | 2.2 | 4.6 |  |
|  | |  | Liver spread | 0.9 |  | 0.5 | 0.2 | 0.3 |  | 1.5 | 1.3 |  |
|  | |  | Meat spread | 2.2 |  | 0.4 | 1.1 | 0.5 |  | 3.9 | 3.9 |  |
|  | | Fresh meat | | 5.1 |  | 2.2 | 0.9 | 4.0 |  | 8.2 | 15.7 |  |
|  | |  | Poultry | 2.4 |  | 0.7 | 0.2 | 2.6 |  | 3.9 | 10.6 |  |
|  | |  | Venison | 0.3 |  | 0.0 | 0.1 | 0.0 |  | 0.6 | 0.6 |  |
|  | |  | Other fresh meat | 2.4 |  | 1.5 | 0.6 | 1.4 |  | 3.7 | 4.5 |  |
|  | | Other meat | | 1.6 |  | 0.1 | 1.3 | 0.2 |  | 2.5 | 3.0 |  |
| Other | | | | 1.2 |  | 1.2 | 1.5 | 1.3 |  | 1.0 | 0.6 |  |
|  | | Sugar and sweets | | 0.7 |  | 0.7 | 1.5 | 1.0 |  | 0.4 | 0.6 |  |
|  | |  | Sugar | 0.0 |  | 0.0 | 0.0 | 0.0 |  | 0.0 | 0.0 |  |
|  | |  | Sweet spread | 0.0 |  | 0.0 | 0.0 | 0.0 |  | 0.0 | 0.0 |  |
|  | |  | Chocolate | 0.6 |  | 0.4 | 1.1 | 0.7 |  | 0.3 | 0.5 |  |
|  | |  | Candy | 0.2 |  | 0.2 | 0.3 | 0.4 |  | 0.1 | 0.1 |  |
|  | | Snacks | | 0.5 |  | 0.6 | 0.1 | 0.2 |  | 0.6 | 0.0 |  |
|  | |  | Chips | 0.0 |  | 0.1 | 0.0 | 0.0 |  | 0.1 | 0.0 |  |
|  | |  | Other snacks | 0.1 |  | 0.1 | 0.0 | 0.0 |  | 0.1 | 0.0 |  |
|  | |  | Nuts and seeds | 0.4 |  | 0.5 | 0.1 | 0.2 |  | 0.5 | 0.0 |  |
| Vegetables | | | | 15.2 |  | 27.4 | 6.7 | 35.8 |  | 12.3 | 0.1 |  |
|  | | Potatoes | | 5.5 |  | 10.7 | 4.4 | 8.3 |  | 3.7 | 0.0 |  |
|  | | Fresh vegetables | | 9.2 |  | 16.0 | 2.2 | 27.1 |  | 8.1 | 0.0 |  |
|  | | Canned vegetables | | 0.5 |  | 0.7 | 0.1 | 0.4 |  | 0.5 | 0.1 |  |
